# Comparative evaluation of Icon® resin infiltration and Clinpro™ XT varnish on colour and fluorescence changes of white spot lesions: a randomized controlled trial

**DOI:** 10.1186/s40510-019-0276-y

**Published:** 2019-06-17

**Authors:** Annapurna Kannan, Sridevi Padmanabhan

**Affiliations:** Department of Orthodontics and Dentofacial Orthopaedics, Faculty of Dental Sciences, Sri Ramachandra Institute of Higher Education and Research, Chennai, India

**Keywords:** White spot lesions, Icon® resin infiltration, Clinpro™ XT varnish

## Abstract

**Background:**

The aim of this trial was to comparatively evaluate Icon® resin infiltration and Clinpro™ XT varnish in restoring aesthetics of white spot lesions (WSLs) present post-orthodontic treatment.

**Materials and methods:**

Two hundred forty WSLs were detected in 193 teeth of 12 patients. The participants were analysed—before intervention (T_0_), immediately after intervention (T_1_), 3 months later (T_2_) and 6 months later (T_3_), with a 1:1 allocation ratio for the application of Icon® resin infiltration and Clinpro™ XT varnish. Using a computer-generated allocation sequence, block randomization was done. A spectrophotometer was used to assess the colour of WSLs and the adjacent enamel, while a DIAGNOdent® was used to assess the fluorescence loss.

**Results:**

Immediately after the intervention, Icon® resin infiltration showed statistically significant better improvement than Clinpro™ XT varnish in restoring the colour (*p* = 0.000); however, at 3 (*p* = 0.001) and 6 months (*p* = 0.000), this was reversed. Except at 3 months, the fluorescence loss sequentially reduced more for Icon® resin infiltration (4.48 ± 1.42 at T_0_ to 1.48 ± 0.81 at T_3_) and was not statistically significant.

**Conclusions:**

Clinpro™ XT varnish showed significantly better improvement than Icon® resin infiltration in restoring the colour and lightness of the WSLs at 3 and 6 months. The fluorescence loss significantly recovered with both intervention methods between immediate application and at 6 months. However, Clinpro™ XT varnish-treated WSLs showed a statistically significant difference compared to the adjacent sound enamel at 6 months.

## Background

Aesthetics is one of the cornerstone objectives of orthodontic treatment. One of the deleterious effects of orthodontic treatment, primarily caused due to poor oral hygiene maintenance, is the occurrence of white spot lesions (WSLs) [1].

The management of WSLs can be divided into prevention (prior to the formation of WSLs), intervention (during the course of orthodontic treatment) and treatment (after the completion of orthodontic treatment). In the prevention and intervention categories, fluorides in the form of varnish, toothpaste, mouthwash, sealant and miswaks [2]; CPP-ACP, NovaMin®; laser therapy; silver nanoparticles; and ozone have been used extensively [3–7]. However, the clinicians often encounter WSLs post-debonding. The effects of the prevention methods are inadequate due to their effectiveness being restricted to only remineralizing the superficial surface of the lesion and not the body of the lesion [8].

The treatment of the WSLs depends on its severity. The mild forms of WSLs can be left to natural remineralization which occurs over a period of 1 year [1, 9] and through other means such as CPP-ACP [3, 10] and fluoride varnishes [11]. The moderate and severe forms can be treated using bleaching, microabrasion, resin infiltration and restorations [12, 13].

The recent emphasis on the mini aesthetics of tooth such as its colour, texture and enamel translucency has created more concern among patients and orthodontists. This augurs an immediate solution.

With Icon® resin infiltration (DMG America, Englewood, NJ, USA), its acid etchant—15% hydrochloric acid—removes the surface layer of the decalcified area due to its penetration depth of 58 ± 37 μm. [14, 15]. This opens up access to the body of the lesion which allows the resin to occlude the pores. The body of the lesion is rendered watertight by means of the resin which has a refractive index (RI Icon® = 1.44) close to that of healthy enamel (RI = 1.63) [14] and also helps in stopping the diffusion of acids by creating a barrier within the lesion and not on the surface [16, 17].

Icon® resin infiltration has been proved to provide immediate restoration of aesthetics of mild WSLs present post-orthodontic treatment to match that of the adjacent sound enamel [18, 19]. It has been shown to remain durable for 6 months [18] with no significant changes at 12 and 24 months [20]. With moderate lesions, a sequential improvement was seen to occur over a period of time [19]. The fluorescence loss of the WSLs significantly recovered immediately with resin infiltration and remained unchanged at the end of 6 weeks in artificially created WSLs [21]. These factors establish Icon® resin infiltration as a gold standard intervention method in aesthetic restoration of white spot lesions.

Resin-modified glass ionomer cement contains fluoroaluminosilicate glass. The fluoride reacts at the surface to provide an immediate release, while the bulk of the glass matrix offers a reservoir of fluoride for sustained release [22, 23] which is said to provide remineralization.

Clinpro™ XT varnish (3M ESPE, Pymble, New South Wales, Australia), an RMGIC product, has been widely used previously for treating dentinal hypersensitivity [24, 25]. In orthodontics, it is shown to be effective in preventing WSLs from occurring during orthodontic treatment [23, 26, 27] and treating artificially induced demineralized areas [28]. However, its efficacy in treating WSLs present post-orthodontic treatment has not yet been tested.

### Specific objective

With both Icon® resin infiltration and Clinpro™ XT varnish, being minimally invasive and site-specific in office single sitting procedures containing composite resin, the aim of this randomized clinical trial was to comparatively evaluate the efficacy of Clinpro™ XT varnish against the ‘gold standard’ Icon® resin infiltration in restoring the aesthetics of the enamel affected by white spot lesions post-orthodontic treatment.

## Materials and methods

The study was conducted at the Department of Orthodontics, Sri Ramachandra Institute of Higher Education and Research after the approval from the University’s Institutional Ethics Committee [number: CSP/16/SEP/51/280 dated October 4, 2016].

### Trial design

This was a parallel-group, randomized, active-controlled trial with a 1:1 allocation ratio for the application of Icon® resin infiltration and Clinpro™ XT varnish.

### Participants, eligibility criteria and settings

Fifty patients who had fixed orthodontic appliances removed during April 2017 were screened for WSLs using Gorelick’s scale [29], and only mild and moderate lesions were considered. This was confirmed using a DIAGNOdent® (KAVO Dental Corporation, Lake, Zurich, IL, USA). Only WSLs having fluorescence loss (*Q*) scores of 2–9 were considered.

### Inclusion criteria

- Patients within 14–30 years of age
- Symmetrical number of permanent teeth in each arch (mesial to second molars)

### Exclusion criteria

- Active carious lesions
- Facial surface restorations
- Deciduous teeth
- Fluorosis
- Intrinsic and extrinsic strains

### Enrollment

A consent form was obtained from those participants willing to be part of the trial.

### Sample calculation

Calculations [colour change (Δ*E*) threshold value = 3.0, *d* = 0.5, *α* error = 0.05 and power of study = 85%] were based on the study by Knosel et al. [18] (219 WSLs; 111 control, 108 treatment) to detect a clinically relevant difference between the two trial arms. This indicated that 115 non-cavitated, unrestored WSLs were required in each arm.

### Randomization

Using a computer-generated programme (www.randomization.com), block randomization allocation sequence [AABB; ABBA; BBAA; BAAB (A-Icon® resin infiltration, B-Clinpro™ XT varnish)] was generated to equally distribute the intervention methods.

A total of 240 non-cavitated, unrestored WSLs after multibracket treatment were detected in 193 teeth of 12 patients [7 females (18 years ± 2 months), 5 males (20 years ± 6 months)] and formed a part of the study.

### Allocation concealment and blinding

The participants were blinded until the allocation of the intervention method. Further blinding was not possible as the intervention methods had different techniques of material application.

### Intervention

All the procedures were performed by the same clinician. On completion of orthodontic treatment, residual composite cleanup and polishing were done.

The VITA Easyshade® advance spectrophotometer (VITA Zahnfabrik, Bad Sackingen, Germany) was used to objectively assess the colour of WSLs and the adjacent sound enamel, using the Munsell system: CIE colour parameters (Δ*E*): *L*, *a* and *b*.

$$ \Delta E={\left(\Delta L\ast {}^2+\Delta a\ast {}^2+\Delta b\ast {}^2\right)}^{\left(1/2\right)} $$

L* refers to the lightness coordinate, and its value ranges from 0 for perfect black to 100 for perfect white. a* and b* are the chromaticity coordinates in the red-green axis and yellow-blue axis, respectively [30, 31].

Similarly, the laser fluorescence method, DIAGNOdent®, was used to assess the fluorescence loss (*Q*) of WSLs and the adjacent sound enamel present around the lesions.

The DIAGNOdent® illuminates the teeth with blue laser light. The tooth dentin contains atoms called fluorophores which fluoresce green when illuminated with blue laser light. When a WSL is present, it appears as a black area surrounded by green reflected colour and this is seen as fluorescence loss [32].

## Intervention methods

### Icon® resin infiltration

First, Icon® Etch, DMG, was applied over the WSLs for 2 min. This was followed by water rinsing and air blowing. Then, Icon® Dry, DMG, was applied for 30 s and air blown. Using the provided sponge applicator, Icon® Infiltrant, DMG, was rubbed on and left for 3 min. Subsequently, it was light-cured for 60 s.

### CLINPRO™ XT varnish

Thirty-seven percent orthophosphoric acid gel was applied for 15 s. On a paper pad, the paste and liquid components were mixed for 10–15 s using an agate spatula and applied over the WSLs. Subsequently, it was light-cured for 20 s.

Both the treatment methods were assessed before intervention (baseline) [T_0_], immediately after intervention [T_1_], 3 months later [T_2_] and 6 months later [T_3_].

Photographs were taken during each time period, and the size and location of the WSLs were mapped at T_0_ for the exactness of the readings at each time interval.

### Outcomes

The primary outcome measure was the difference in colour and fluorescence values between the treatment groups over a period of 6 months, and the secondary outcome measure was the difference within the treatment groups.

### Statistical analysis

The data was analysed with IBM.SPSS statistics software, version 23.0. Kolmogorov-Smirnov’s test was used to check the normality of the data. To find the significant difference within the groups, at different time intervals, a one-way repeated measures ANOVA was used followed by post hoc Tukey test. Between the groups, independent Student’s *t* test was used for the intergroup comparison.

### Error of method (Table 1)

The L*a*b* values of one white spot lesion each in the upper and lower arch of one patient were recorded in vivo three times on three consecutive days. The ranges of variance due to measurement error for *L* value were 0.20 units for the WSL on the maxillary tooth and 0.16 units for WSL on the mandibular tooth. This indicates precision in measurements recorded with spectrophotometer when compared to previous results [33]. All measurements were carried out under normalized conditions to make certain their accuracy and reproducibility.

**Table 1:** Error assessment results

Parameter	Valid (*n*)	Mean	Minimum	Maximum	Range	SD
WSL on the maxillary tooth						
*L*	3	78.54	78.44	78.64	0.20	0.068
*a*	3	0.86	0.47	1.25	0.78	0.258
*b*	3	18.33	18.12	18.54	0.42	0.149
WSL on the mandibular tooth						
*L*	3	74.45	74.37	74.53	0.16	0.048
*a*	3	0.92	0.52	1.32	0.80	0.293
*b*	3	16.69	16.48	16.90	0.42	0.131

*WSL* white spot lesions, *L* lightness, *a* red-green axis, *b* yellow-blue axis

## Results

### Baseline data and recruitment (Fig. 1)

One hundred twenty-four white spot lesions present in 102 teeth of 6 patients received the allocated intervention method of Icon® resin infiltration, while 116 white spot lesions present in 91 teeth of 6 patients received the allocated intervention method of Clinpro™ XT varnish. At T_3_, 1 patient under the Clinpro™ XT varnish intervention method was lost to follow-up.

**Fig. 1:**
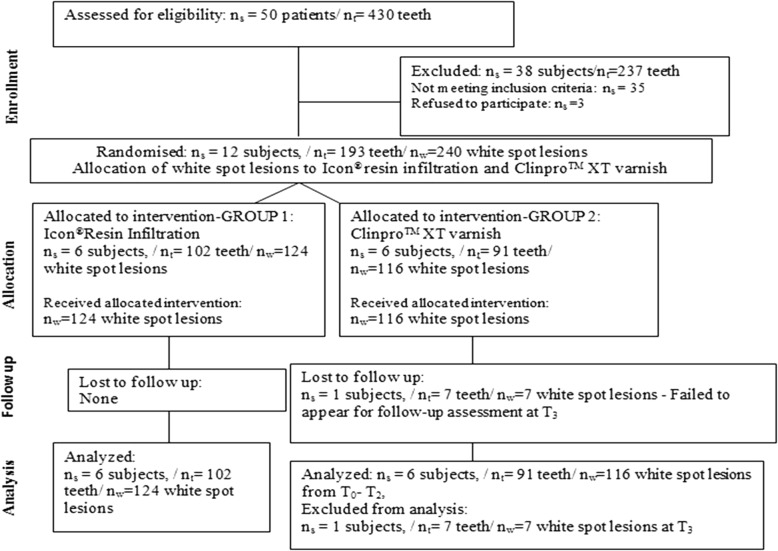
Participant flow and numbers analysed

### Outcomes and estimation

#### Analysis of lightness (*L*) values

##### Intergroup comparison (Table 2).

- Clinpro™ XT varnish showed more increase in the *L* values compared with Icon® resin infiltration, which was statistically significant at T_2_ and T_3._

**Table 2:** Intergroup and intragroup comparison of lightness values at various time intervals

	*L* values of Icon® resin infiltration	l values of Clinpro™ XT varnish	Intergroup comparison	Intragroup comparison	
				Icon® resin infiltration	Clinpro™ XT varnish
T_0_	73.60 ± 7.71	74.62 ± 7.33	*p* = 0.295	T_0_ vs T_1_, *p* = 0.000**	T_0_ vs T_1_, *p* = 0.000**
T_1_	78.37 ± 5.94	78.93 ± 5.40	*p* = 0.446	T_1_ vs T_2_,*p* = 0.211	T_1_ vs T_2_, *p* = 0.003**
T_2_	80.08 ± 6.46	81.68 ± 5.55	*p* = 0.041*	T_2_ vs T_3_, *p* = 0.168	T_2_ vs T_3_, *p* = 0.069
T_3_	81.88 ± 6.27	83.69 ± 5.29	*p* = 0.017*	T_0_ vs T_3_, *p* = 0.000**	T_0_ vs T_3_, *p* = 0.000**
Adjacent sound enamel	81.67 ± 4.69	82.73 ± 4.66	*p* = 0.117	T_3_ vs adjacent sound enamel, *p* = 0.999	T_3_ vs adjacent sound enamel, *p* = 0.761

Statistical Tests: intergroup comparison—independent Student’s *t* test; intragroup comparison—one-way repeated measures ANOVA

*L* lightness, *T*_*0*_ before intervention, *T*_*1*_ immediate after intervention, *T*_*2*_ 3 months, *T*_*3*_ 6 months

*p < 0.05, **p < 0.01

##### Intragroup comparison (Table 2)

- In Icon® resin infiltration and Clinpro™ XT varnish, a statistically significant increase in the *L* value was seen from T_0_ to T_1_.
- In both groups, a statistically significant overall improvement was seen in the *L* value from T_0_ to T_3_.

#### Analysis of colour changes (Δ*E*) (Table 3)

- Icon® resin infiltration showed a statistically significant colour change value from T_0_ to T_1_ (ΔE_1_) than Clinpro™ XT varnish.
- Clinpro™ XT varnish showed a statistically significant colour change value from T_0_ to T_2_ (Δ*E*_2_) and from T_0_ to T_3_ (Δ*E*_3_) than Icon® resin infiltration.

**Table 3:** *a* and *b* values and intergroup comparison of colour changes at various time intervals

	Adjacent sound enamel		T_0_		T_1_		Δ*E*_1_ (T_0_–T_1_)	T_2_		Δ*E*_2_ (T_0_–T_2_)	T_3_		Δ*E*_3_ (T_0_–T_3_)
	*a*	*b*	*a*	*b*	*a*	*b*		*a*	*b*		*a*	*b*	
Icon® resin infiltration	0.20 ± 1.54	26.30 ± 7.78	0.026 ± 1.76	18.39 ± 5.31	− 0.29 ± 1.38	21.47 ± 7.32	5.68 ± 1.24	− 0.37 ± 1.18	21.41 ± 6.11	7.19 ± 1.17	− 0.57 ± 1.71	23.34 ± 10.93	9.66 ± 1.42
Clinpro™ XT varnish	− 0.37 ± 1.14	24.08 ± 5.85	− 0.51 ± 1.41	17.71 ± 4.08	− 0.58 ± 1.23	19.58 ± 6.89	4.61 ± 1.13	− 0.63 ± 1.06	20.74 ± 6.66	7.68 ± 1.12	− 1.03 ± 1.49	23.74 ± 6.66	10.59 ± 1.21
Inter group comparison of colour changes							0.000**			0.001**			0.000**

Statistical tests: intergroup comparison—independent Student’s *t* test

*a* red-green axis, *b* yellow-blue axis, *ΔE* colour changes, *T*_*0*_ before intervention, *T*_*1*_ immediate after intervention, *T*_*2*_ 3 months, *T*_*3*_ 6 months

**p < 0.01

#### Analysis of fluorescence loss (*Q*)

##### Intergroup comparison (Table 4).

- At all other time points except T_2_, *Q* value reduction was seen more with Icon® resin infiltration when compared to Clinpro™ XT varnish. However, on comparison, it was not significant.

**Table 4:** Intergroup and intragroup comparison of fluorescence loss (*Q*) values at various time intervals

	*Q* values of Icon® resin infiltration	*Q* values of Clinpro™ XT varnish	Intergroup comparison	Intragroup comparison	
				Icon® resin infiltration	Clinpro™ XT varnish
T_0_	4.48 ± 1.42	4.60 ± 1.29	*p* = 0.498	T_0_ vs T_1_, *p* = 0.000**	T_0_ vs T_1_, *p* = 0.000**
T_1_	2.84 ± 1.53	3.14 ± 1.20	*p* = 0.094	T_1_ vs T_2_, *p* = 0.000**	T_1_ vs T_2_, *p* = 0.000**
T_2_	2.06 ± 1.05	2.05 ± 0.83	*p* = 0.935	T_2_ vs T_3_, *p* = 0.000**	T_2_ vs T_3_, *p* = 0.000**
T_3_	1.48 ± 0.81	1.51 ± 0.72	*p* = 0.767	T_0_ vs T_3_, *p* = 0.000**	T_0_ vs T_3_, *p* = 0.000**
Adjacent Sound Enamel	1.06 ± 0.58	1.08 ± 0.51	*p* = 0.80	T_3_ adjacent sound enamel, *p* = 0.050	T_3_ adjacent sound enamel, *p* = 0.016*

Statistical tests: intergroup comparison—independent Student’s *t* test; intragroup comparison—one-way repeated measures ANOVA

*Q* fluorescence loss, *T*_*0*_ before intervention, *T*_*1*_ immediate after intervention, *T*_*2*_ 3 months, *T*_*3*_ 6 months

*p < 0.05, **p < 0.01

##### Intragroup comparison (Table 4).

- In both groups, a statistically significant fluorescence loss amelioration was seen from T_0_ to T_1_ to T_2_ to T_3_.
- At T_3_, in both groups, the amelioration of the fluorescence loss brought the values close to that of the adjacent sound enamel, which was not statistically significant for Icon® resin infiltration but statistically significant for Clinpro™ XT varnish.

## Discussion

A parallel-group trial as opposed to a split study design was carried out to avoid any treatment bias to mitigate any carry-across effect of fluorides present in Clinpro™ XT varnish. [34–36]

The difference in the refractive index of the enamel and lesions’ crystals contributes to the whitish nature of the WSLs [37]. WSLs have lower L* values due to the large portions of transmitted light being absorbed and scattered within the micropores of the body of the lesion [38–40]. Hence, the lightness value is of vital importance.

As expected, following the application of Icon® resin infiltration, a significant increase was seen immediately in the lightness of the WSLs, suggestive of an immediate increase in enamel reflectivity (T_1_). The subsequent increase from T_1_ to T_3_ was not statistically significant, showing the durable nature of the material (Table 2).

Surprisingly with Clinpro™ XT varnish, a significant increase was seen immediately in the lightness of the WSLs with its application. It can be hypothesized that the usage of 37% orthophosphoric acid would have penetrated and removed the surface layer of the WSL, opening up access to the body of the lesion. The PC would have further increased with the usage of low viscous resin HEMA. The presence of Bis-GMA could have enhanced the resin reactivity and reflectivity, aiding in the immediate improvement in the optical properties of the WSLs. At T_2_, a significant increase was further seen in the lightness and this could be attributed to the release of fluoride from the fluoroaluminosilicate glass particles. The small amount of increase seen from T_2_ to T_3_ was probably due to the sustained release of fluoride and calcium glycerophosphate from the reservoir of the glass matrix. Further, the overall change seen with Clinpro™ XT was significantly greater than Icon® resin infiltration (Table 2).

The resultant Δ*E* values calculated at all time intervals for both groups were more than 3.7 Δ*E* units, which is the critical value for clinical detection [41] and were statistically significant. However, the comparison between the groups showed that Icon® resin infiltration demonstrated a significant immediate improvement, whereas the long-term improvement was more significant with Clinpro™ XT varnish (Table 3).

Icon® resin infiltration showed more revival of the lost fluorescence at T_1_ than Clinpro™ XT, probably due to the creation of an immediate diffusion barrier within the body of the lesion. At T_2_, a comparable improvement was seen with both groups. At T_3_, Icon® resin infiltration showed further amelioration of the fluorescence loss than Clinpro™ XT, though not statistically significant. Both groups at T_3_ had a *Q* value lesser than that of the incipient demineralization value range of 2–9 [42]. Yet, the results have to be cautiously interpreted, as Icon® resin infiltration only occludes the acid pathways [15, 43] and does not remineralize the WSLs per se (Table 4).

**Fig. 2:**
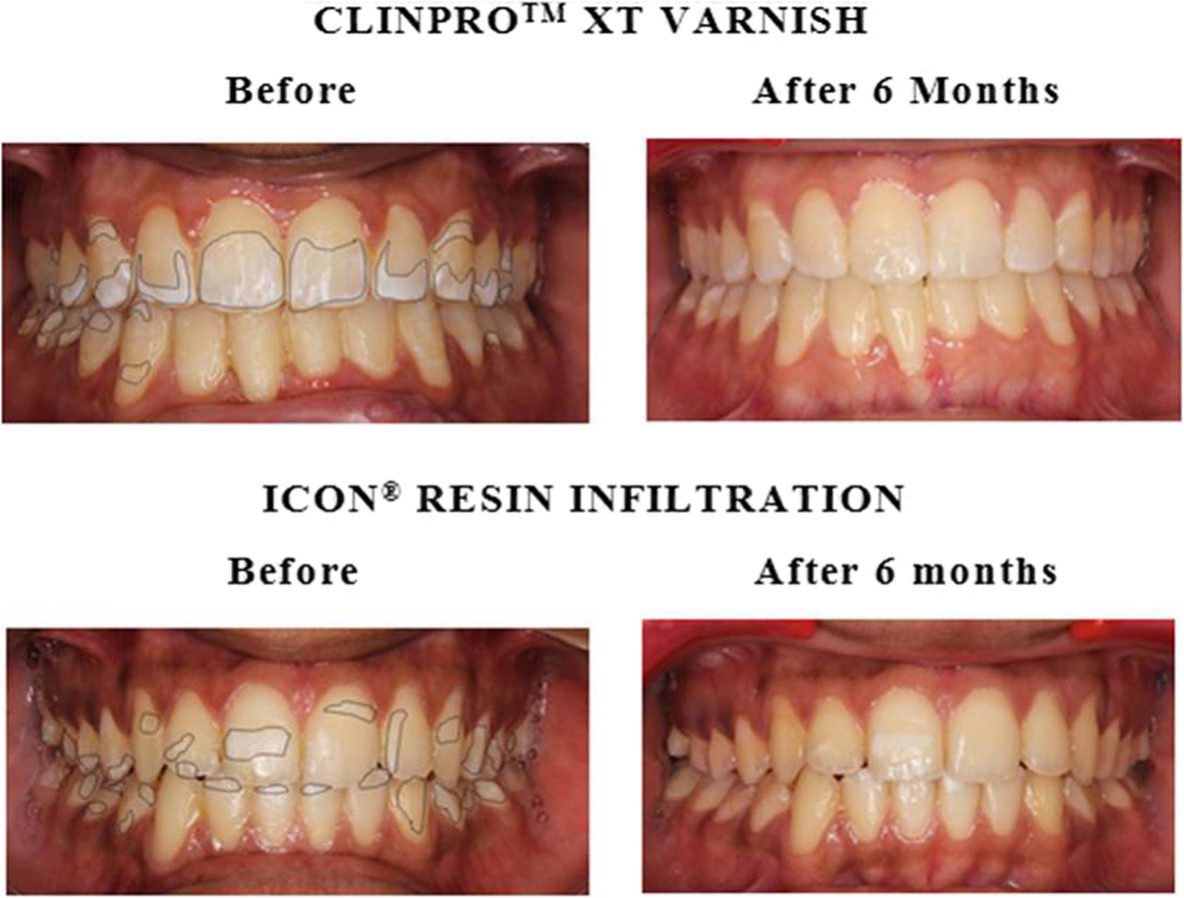
Comparison of interventions

Clinically, repeated etching was required with Icon® resin infiltration for moderate lesions to provide a preview of the aesthetic result to be expected after infiltration. Both Icon® resin infiltration and Clinpro™ XT varnish provided immediate aesthetic results with mild lesions. However, with moderate lesions, though progressive improvements were seen in both groups, clinically, visible improvements were seen more with Clinpro™ XT at T_3_ than with Icon® resin infiltration (Fig. 2).

While both methods showed a significant increase in improving the aesthetics of the WSLs, the enamel in response to Clinpro™ XT was more comparable to that of the adjacent sound enamel. Icon® resin infiltration has already been proven in restoring the aesthetics of the enamel, and this study offers Clinpro™ XT varnish as an alternative with gratifying short- as well as long-term results.

Further, long-term follow-up is required to see the effects of Clinpro™ XT varnish on moderate WSLs over a year’s period to see if the colour and fluorescence are completely restored.

## Conclusion

- Immediately after the intervention, Icon® resin infiltration demonstrated a significantly better improvement than Clinpro™ XT varnish in restoring the colour; however, at 3 and 6 months, this was reversed.
- At the end of the study period, the fluorescence loss significantly ameliorated in response to both Icon® resin infiltration between immediate intervention and at 6 months.
- However, at 6 months, the fluorescence of the white spot lesions infiltrated with Icon® resin was comparable to that of the adjacent sound enamel while those treated with Clinpro™ XT varnish showed a statistically significant difference.

## Abbreviations

Δ*E*: Colour changes
*L*: Lightness
*Q*: Fluorescence loss
WSLs: White spot lesions
