# Correction to: Comparative evaluation of Icon® resin infiltration and Clinpro™ XT varnish on colour and fluorescence changes of white spot lesions: a randomized controlled trial

**DOI:** 10.1186/s40510-019-0283-z

**Published:** 2019-07-26

**Authors:** Annapurna Kannan, Sridevi Padmanabhan

**Affiliations:** Department of Orthodontics and Dentofacial Orthopaedics, Faculty of Dental Sciences, Sri Ramachandra Institute of Higher Education and Research, Chennai, India

**Correction: Prog Orthod (2019) 20:23**

**https://doi.org/10.1186/s40510-019-0276-y**

In the publication of this article [1], there is an error in the Materials and methods section.

The error:

“The study was conducted at the Department of Orthodontics, XXX: Sri Ramachandra Institute of Higher Education and Research after the approval from the University’s Institutional Ethics Committee [number: CSP/16/SEP/51/280 dated October 4, 2016].

Should instead read:

The study was conducted at the Department of Orthodontics, Sri Ramachandra Institute of Higher Education and Research after the approval from the University’s Institutional Ethics Committee [number:CSP/16/SEP/51/280 dated October 4, 2016].

This has now been updated in the original article [1].
